# Genetic diversity of Italian goat breeds assessed with a medium-density SNP chip

**DOI:** 10.1186/s12711-015-0140-6

**Published:** 2015-08-04

**Authors:** Letizia Nicoloso, Lorenzo Bomba, Licia Colli, Riccardo Negrini, Marco Milanesi, Raffaele Mazza, Tiziana Sechi, Stefano Frattini, Andrea Talenti, Beatrice Coizet, Stefania Chessa, Donata Marletta, Mariasilvia D’Andrea, Salvatore Bordonaro, Grazyna Ptak, Antonello Carta, Giulio Pagnacco, Alessio Valentini, Fabio Pilla, Paolo Ajmone-Marsan, Paola Crepaldi, the Italian Goat Consortium

**Affiliations:** DIVET, Università degli Studi di Milano, via Celoria 10, 20133 Milan, Italy; Istituto di Zootecnica, Università Cattolica del Sacro Cuore, via Emilia Parmense, 84, 29122 Piacenza, Italy; Associazione Nazionale della Pastorizia, via Palmiro Togliatti 1587, 00155 Rome, Italy; Laboratorio Genetica e Servizi (LGS) - Associazione Italiana Allevatori (AIA), via Bergamo, 292, 26100 Cremona, Italy; Agris Sardegna, Unità di Ricerca di Genetica e Biotecnologie, Sassari, Italy; CNR – IBBA, UOS di Lodi, via Einstein, Località Cascina Codazza, 26900 Lodi, Italy; Dipartimento di Agricoltura, Alimentazione e Ambiente Di3A, Università degli Studi di Catania, via Valdisavoia 5, 95123 Catania, Italy; Dipartimento Agricoltura, Ambiente e Alimenti, Università degli Studi del Molise, via Francesco De Sanctis s.n.c., 86100 Campobasso, Italy; Dipartimento Scienze Biomediche Comparate, Università di Teramo, Piazza Aldo Moro 45, Teramo, Italy; Dipartimento per l’Innovazione nei sistemi Biologici, Agroalimentari e Forestali, Università della Tuscia, via de Lellis, 01100 Viterbo, Italy

## Abstract

**Background:**

Among the European countries, Italy counts the largest number of local goat breeds. Thanks to the recent availability of a medium-density SNP (single nucleotide polymorphism) chip for goat, the genetic diversity of Italian goat populations was characterized by genotyping samples from 14 Italian goat breeds that originate from different geographical areas with more than 50 000 SNPs evenly distributed on the genome.

**Results:**

Analysis of the genotyping data revealed high levels of genetic polymorphism and an underlying North–south geographic pattern of genetic diversity that was highlighted by both the first dimension of the multi-dimensional scaling plot and the Neighbour network reconstruction. We observed a moderate and weak population structure in Northern and Central-Southern breeds, respectively, with pairwise F_ST_ values between breeds ranging from 0.013 to 0.164 and 7.49 % of the total variance assigned to the between-breed level. Only 2.11 % of the variance explained the clustering of breeds into geographical groups (Northern, Central and Southern Italy and Islands).

**Conclusions:**

Our results indicate that the present-day genetic diversity of Italian goat populations was shaped by the combined effects of drift, presence or lack of gene flow and, to some extent, by the consequences of traditional management systems and recent demographic history. Our findings may constitute the starting point for the development of marker-assisted approaches, to better address future breeding and management policies in a species that is particularly relevant for the medium- and long-term sustainability of marginal regions.

**Electronic supplementary material:**

The online version of this article (doi:10.1186/s12711-015-0140-6) contains supplementary material, which is available to authorized users.

## Background

According to archeozoological and genetic data, goats were domesticated some 10 000 years ago in the geographical region that spans from Eastern Anatolia to the Zagros Mountains in Northern Iran [1, 2]. After domestication, goats quickly spread all over the world following human migrations and commercial trade [1]. They rapidly adapted to a very wide range of environmental conditions and started to play economic, cultural and religious roles in many human cultures. Today, goats represent an important source of milk, meat and fiber (e.g., cashmere wool) especially in marginal rural areas, dry lands and mountains, particularly in developing countries. As a consequence of an increase in farmland abandonment in marginal areas, the genetic diversity of many goat populations is being rapidly eroded or lost, particularly in Europe that counts 200 recorded goat breeds (according to the Food and Agriculture Organization (FAO) [3].

In modern European agriculture, the economic role of goats is mainly linked to the products of either high-yielding dairy breeds that developed in the central Alps (e.g., the Saanen and Toggenburg breeds of Swiss origin) or of local stocks that were often improved by crossing with more productive dairy or meat breeds (e.g., cosmopolitan Boer). In Europe, 96 % of the 2.8 millions of tons of goat products are dairy products and only 4 % are meat products (see FAOSTAT at faostat.fao.org).

Even if the main hotspots of the world goat diversity are probably in Africa and Asia, among European countries, Italy can be considered as a reservoir of genetic resources for the caprine species with 36 breeds recorded by the National Goat and Sheep Breeder Association (www.assonapa.it). Diversity in orography and climate together with historical factors and traditions, led to the development of a large variety of livestock populations, which were later standardized in modern breeds. Although, since the early 1950s the genetic diversity of farm species has suffered from steady erosion, an opposite trend is observed for goats. A number of local goat breeds still populate rural environments where harsh climate and pastures challenge the diffusion of more productive species and cosmopolite breeds. According to the FAO classification, 58.3 % of Italian goat breeds are considered not to be at risk, each with more than 1200 heads, but there is a declining trend. These breeds include three cosmopolite or exotic breeds (Saanen, Camosciata delle Alpi and Maltese), which represent 24 % of recorded Italian goat heads. Among the remaining 41.7 % of breeds classified as at risk, 11 are endangered (number of heads less than 1200 with a declining trend) including Orobica, Valdostana and Ciociara Grigia breeds, and four are in a critical status (number of heads less than 100) as for example the Di Teramo breed.

Italian goats are mainly reared in the Alps and in the Mediterranean environments that are typical of Southern Italy and of the islands. In 2013, the regions that counted the highest proportion of goats were Calabria (43 % of animals), the two major islands Sardinia (18 %) and Sicily (10 %) in Southern Italy, and Lombardy (10 %) in the north (www.assonapa.com). These data from the national goat and sheep breeders association refer only to registered animals that, according to FAOSTAT, represent only 20 % of the goats reared in Italy.

Goat farming systems in Italy vary widely depending on region and on the breeds raised: in the north, two main different farming systems are present: (1) traditional farming, with an indoor system in the winter, natural grazing in the spring and autumn, and vertical transhumance in the summer; (2) intensive and semi-intensive indoor farming, with animals kept in flocks of medium to large size and reared under controlled feeding that includes hay and concentrate. The farming system for breeds in Central Italy is characterized by small sedentary herds that practice transhumance from spring to fall while that for breeds in Southern Italy is mainly characterized by small- to large-sized farms with either semi-sedentary farming based on natural pasture or free-ranging farming. In Sardinia, goat farming systems differ from the traditional ones and range from small infrastructures with low management costs to a semi-intensive system as for Maltese goats [4].

Several molecular studies, which often included limited numbers of breeds and loci, have been carried out on local Italian goat breeds [5–7] in attempts to monitor genetic erosion and identify conservation strategies. Large-scale surveys based on nuclear markers, such as microsatellites [8], AFLP (amplified fragment length polymorphisms) [7] or small panels of SNPs (single nucleotide polymorphisms) [9–11], detected a remarkable level of genetic diversity in European goats. Recently, a meta-analysis of worldwide goat microsatellite datasets highlighted a decreasing gradient of diversity from the domestication centre towards Europe and Asia, and a clear phylogeographic structure at both the continental and regional levels [12]. In particular, breed formation seems to have been less systematic in the Middle East than in North-Central Europe, where several breeds are recognised as separate gene pools, partly as a consequence of inbreeding and partly of a strong genetic identity [9]. On the contrary, results obtained from mitochondrial DNA analyses [13] revealed a weak phylogeographic structure, which suggested a long-lasting intercontinental gene flow as a consequence of the frequent translocation of goats along colonization, migration and commercial routes [14].

Recently, the availability of SNP panels [15–17] allows the investigation of livestock genomic diversity at a level of resolution that is impossible to reach with other types of markers. In this study, we exploited the medium-density (>50 000 SNPs) BeadChip available for goat [18] to assess the genome-wide diversity of 14 Italian goat breeds, as a contribution to biodiversity conservation and prioritisation actions.

## Methods

### Biological samples

**Fig. 1:**
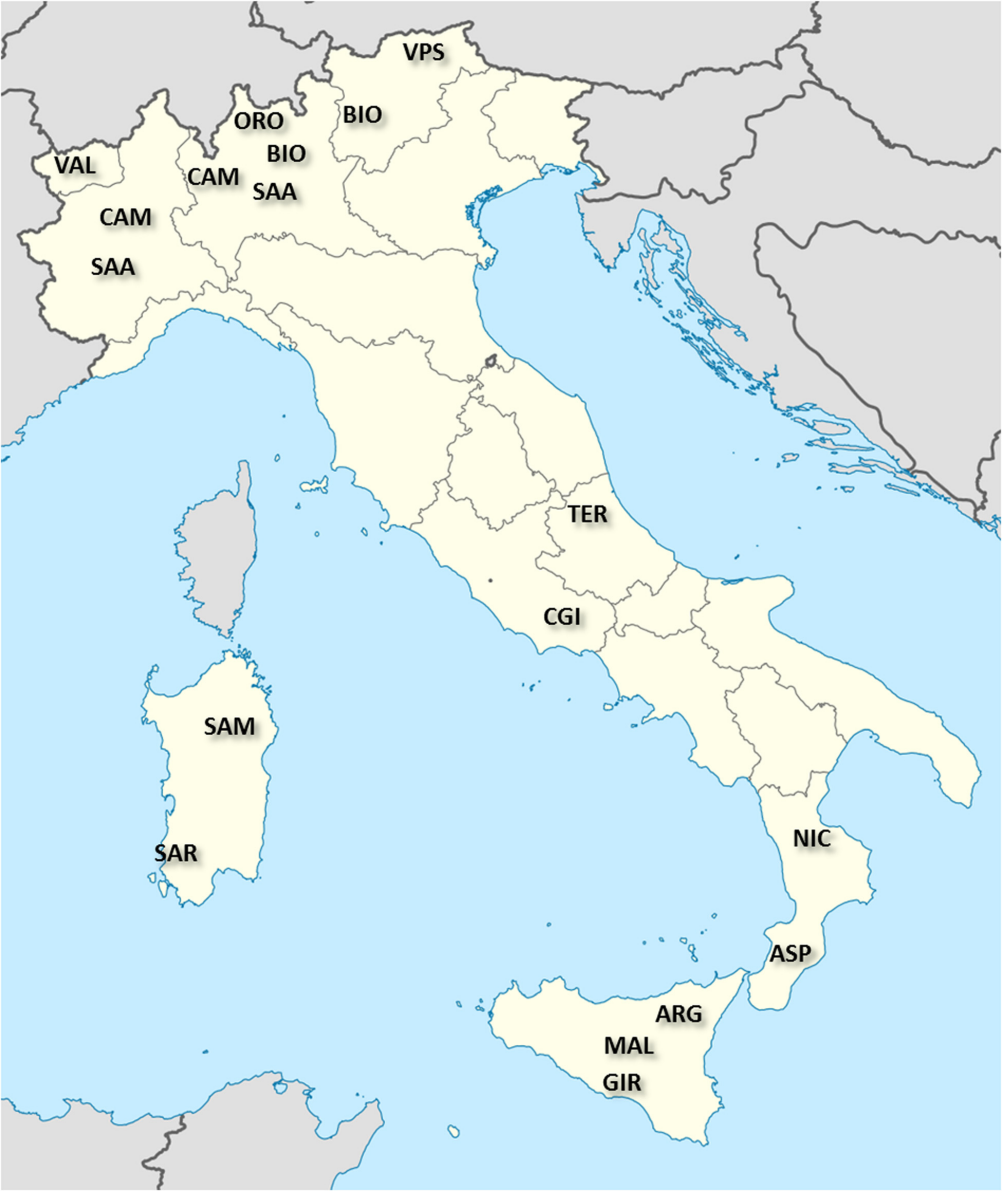
Geographic origin of the analyzed Italian goat breeds

A total of 354 animals from 14 Italian goat breeds were sampled (Table 1): Bionda dell’Adamello, BIO, *n* = 24; Camosciata delle Alpi, CAM, *n* = 31; Orobica, ORO, *n* = 24; Saanen, SAA, *n* = 24; Valpassiria or Passeirer Gebirgziege, VPS, *n* = 24; Valdostana, VAL, *n* = 24; Ciociara Grigia, CGI, *n* = 19; Dell’Aspromonte, ASP, *n* = 24; Nicastrese, NIC, *n* = 25; Girgentana, GIR, *n* = 24; Argentata dell’Etna, ARG, *n* = 25; Maltese sampled in Sicily, MAL, *n* = 16; Di Teramo, TER, *n* = 23; Sarda, SAR, *n* = 32; Maltese sampled in Sardinia, SAM, *n* = 15 (the Maltese breed was sampled from two geographical areas where it is reared). The geographical origin of the breeds is shown in Fig. 1. To ensure the representativeness of sampling, for each breed, minimally related animals were selected from different farms across the traditional rearing area. The only exception was the TER breed for which the 23 selected animals did not comply with the criterion of minimal relatedness. This population, in fact, was already reported in the Endangered Breeds List of the 3rd edition of World Watch List for domestic animal diversity (year 2000, [19]) with only about 100 to 500 remaining individuals, and currently, it survives on only one farm with about 50 animals.

**Table 1:** Name of the breeds, breed acronyms, sample size before (n-PreQC) and after (n-PostQC) genotyping quality control procedures, expected heterozygosity (H_E_), observed heterozygosity (H_O_), Wright’s inbreeding coefficient (F_IS_), and proportion of polymorphic SNPs (P_N_)

Breed name	Breed code	n-PreQC	n-PostQC	H_E_	H_O_	F_IS_	P_N_
Valdostana	VAL	24	24	0.37	0.36	0.05	98.20
Camosciata delle Alpi	CAM	31	30	0.40	0.40	0.02	99.70
Saanen	SAA	24	24	0.41	0.41	−0.001	99.66
Orobica	ORO	24	23	0.35	0.35	0.01	96.89
Bionda dell’Adamello	BIO	24	24	0.40	0.40	0.02	99.45
Valpassiria or Passeirer Gebirgziege	VPS	24	24	0.40	0.40	0.02	99.41
Ciociara Grigia	CGI	19	19	0.40	0.39	0.06	99.29
Di Teramo	TER	23	23	0.35	0.38	−0.06	95.13
Dell’Aspromonte	ASP	24	24	0.40	0.38	0.06	99.38
Nicastrese	NIC	25	24	0.40	0.38	0.07*	99.37
Argentata dell’Etna	ARG	25	24	0.41	0.41	0.02	99.63
Girgentana	GIR	24	24	0.36	0.36	0.004	96.55
Maltese from Sicily	MAL	16	16	0.37	0.36	0.06	97.87
Sarda	SAR	32	32	0.41	0.39	0.06	99.64
Maltese from Sardinia	SAM	15	15	0.36	0.36	0.02	96.76

^*^ *P* < 0.05

Blood samples were collected according to the recommendations of the European Council (1986) concerning animal care. Whole blood was collected in Vacutainer tubes with K-EDTA as anticoagulant and stored at −20 °C until genomic DNA was extracted using a commercial kit (NucleoSpin Blood, Macherey-Nagel) according to the manufacturer’s instructions.

### Genotyping and SNP quality control

DNA samples were genotyped using the GoatSNP50 BeadChip (Illumina Inc., San Diego, CA) developed by the International Goat Genome Consortium (IGGC) [18]. SNP typing was outsourced at the Associazione Italiana Allevatori - Laboratorio di Genetica e Servizi (http://www.lgscr.it) and Porto Conte Ricerche s.r.l. (Alghero, Sassari, Italy) facilities. Raw signal intensities of the 53 347 SNPs were converted into genotype calls with GenomeStudio software v2011.1 and by using the SNP genomic locations and cluster files made available by the IGGC. GenABEL ver. 1.7-6 [20] was used for quality control (QC) procedures with standard thresholds i.e., a SNP call rate greater than 0.95, a MAF (minor allele frequency) greater than 0.01 and an individual genotype call rate greater than 0.95.

### Data analysis

Data parsing, recoding and formatting were performed using PLINK ver. 1.07 suite [21]. Expected (H_E_) and observed heterozygosities (H_O_) were calculated with an in-house script. Arlequin software ver. 3.5.1.3 [22] (http://cmpg.unibe.ch/software/arlequin3) was used to (i) calculate population specific inbreeding coefficients (F_IS_); (ii) compute the F_ST_ [23, 24] distance matrix between breeds; and (iii) perform an Analysis of MOlecular VAriance (AMOVA, [25]) at different hierarchical levels to test the differentiation between breeds and between groups of breeds from distinct geographical areas (i.e., Northern Italy, Central Italy, Southern Italy and islands).

A Neighbor-network graph based on between-breeds F_ST_ distances was obtained with the software SplitsTree ver. 4.10 [26]. The R package GenABEL ver. 1.7-6 [20] was used to build a multi-dimensional scaling (MDS) plot, based on a matrix of (1-pairwise genomic kinship) distances between individuals. ADMIXTURE ver. 1.22 [27] software was used for population structure analysis with a number of hypothetical pseudo-populations, K, that ranged from 2 to 25. To evaluate optimal partitioning, cross-validation (CV) error values were computed for each K using a 5-fold cross-validation procedure.

## Results

After quality control: (i) 551 markers with a MAF less than 0.01 and (ii) 1660 SNPs and four animals with call rates less than 0.95 were excluded. The final working dataset included 350 animals and 51 136 SNPs of which only 0.46 % carried rare alleles (0.01 ≤ MAF ≤ 0.05).

The percentage of within-breed polymorphic SNPs ranged from 95.13 % to 99.70 % (Table 1) with the highest values found for the SAA and CAM breeds (99.66 % and 99.70 %, respectively), which were both included in the SNP chip discovery panel and the lowest value found for the TER breed (95.13 %). Expected and observed heterozygosities ranged from 0.37 (VAL) to 0.41 (ARG) and from 0.36 (VAL) to 0.41 (SAA), respectively (Table 1). The only significant deviation from Hardy-Weinberg equilibrium was detected in the NIC breed, for which an inbreeding coefficient (F_IS_) of 0.074 (*P* < 0.05) revealed a slight excess of homozygotes. The pairwise F_ST_ values between breeds ranged from 0.013 to 0.164 [See Additional file 1: Table S1]. As a general trend, the ORO breed from Northern Italy and the TER breed from the centre of the peninsula showed the highest F_ST_ values, while ARG, NIC, CGI and ASP breeds from Southern and Central Italy showed the lowest F_ST_ values.

**Fig. 2:**
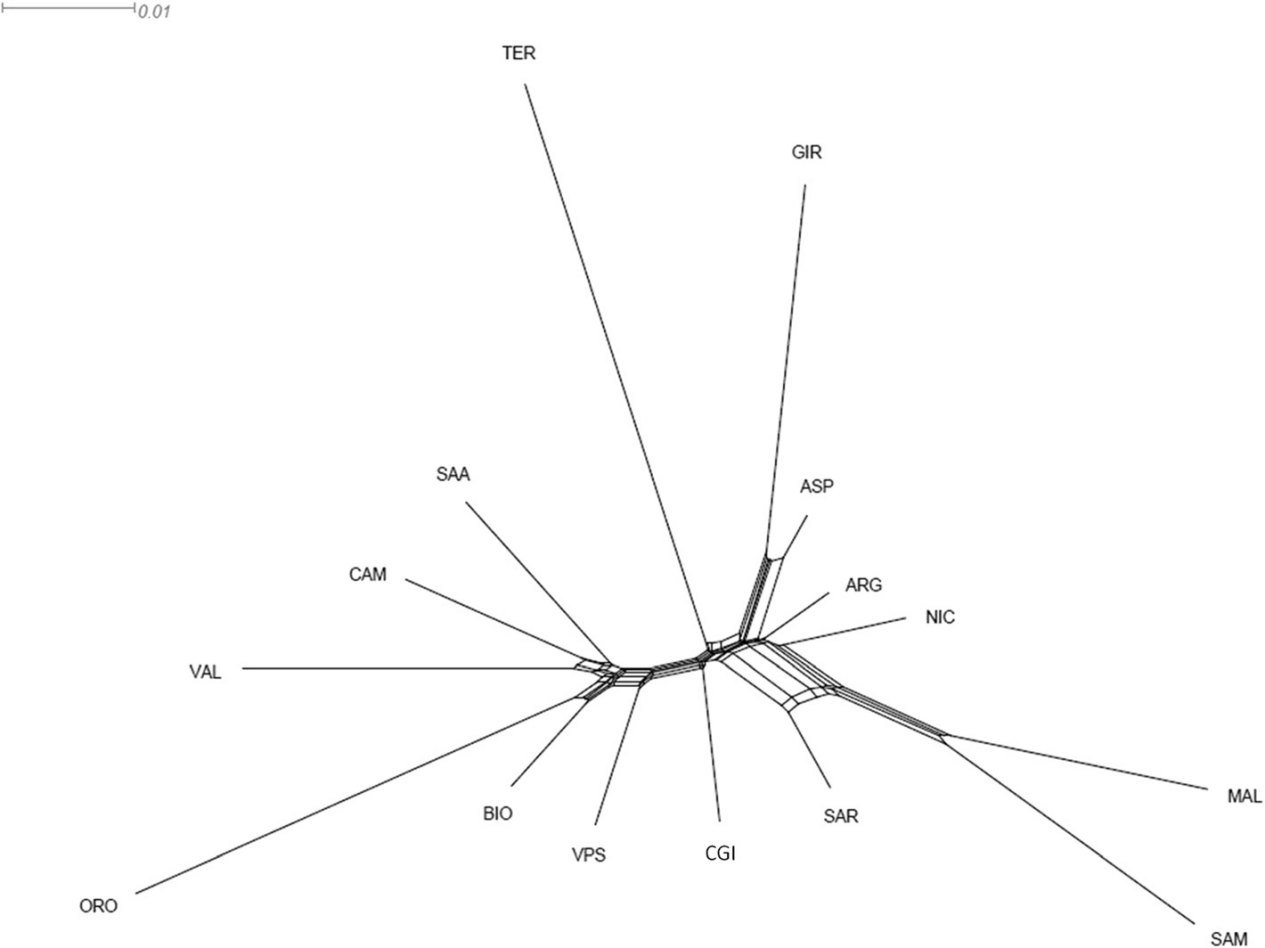
Neighbor-network based on pairwise F_ST_ genetic distances between breeds

The AMOVA analysis [See Additional file 2: Table S2] assigned 7.49 % of the total variance to the between-breed level, while only 2.11 % of the variance explained the clustering of breeds into geographical groups (Northern Italy, Central Italy, Southern Italy and islands). The Neighbour-Network graph (Fig. 2) also revealed the presence of an underlying geographical pattern of variation with the clusters of breeds from left to right corresponding to Northern, Central and Southern Italy. Breeds that shared close genetic relationships were placed on different branches that originated from the same basal node i.e., the two MAL populations that were sampled separately in Sicily and Sardinia, the GIR and ASP, VAL and the CAM, ORO and BIO breeds. Relevant reticulate connections were highlighted only between SAR, MAL and SAM breeds.

**Fig. 3:**
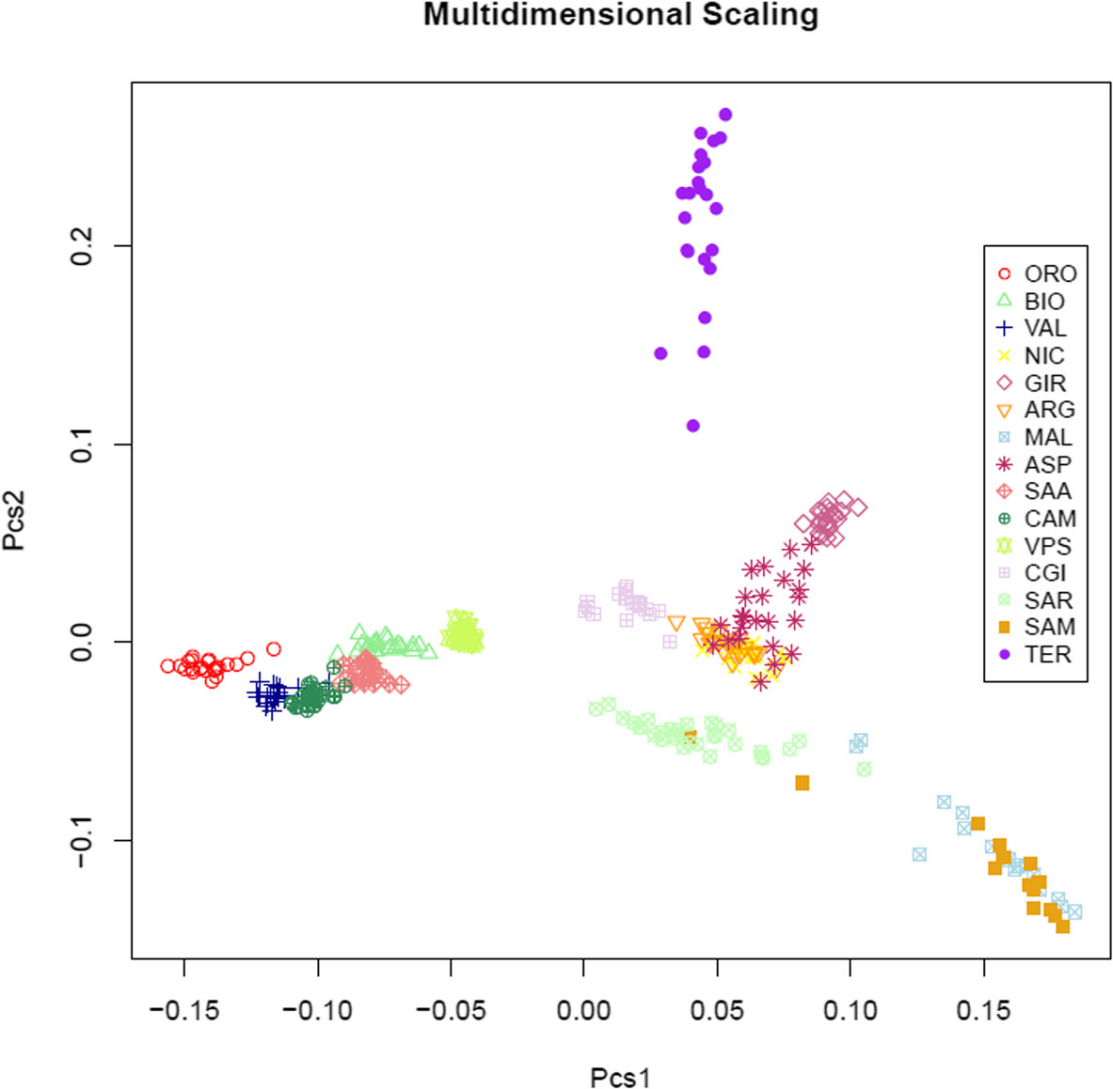
Multidimensional-scaling plot. Multidimensional-scaling plot of distances based on a genomic kinship matrix. The axes corresponding to first (abscissa, variance explained: 6.29 %) vs. second dimension (ordinate, variance explained: 3.88 %) are shown

The first dimension (X axis in Fig. 3, 6.29 % of explained variance) of the MDS plot confirmed the same geographical clustering, while the second dimension (Y axis in Fig. 3, 3.88 % of explained variance) contributed mainly to separate TER and the two Maltese populations from the other breeds. According to the relative position and to the width of the scatter of points for the different breeds, the Northern Italian populations formed a well-defined group, which was clearly separated from the remaining populations by a large gap on the first axis. Breeds from Northern Italy, except for ORO and VPS, overlapped each other, with individual points occupying small areas on the graph, which may account for the reduced within-breed variability, together with some degree of between-breed differentiation. Conversely, among Central and Southern Italian breeds, CGI and GIR were separated in compact and well-defined clusters, while NIC and ARG completely overlapped each other. The coordinates of points for the SAR breed on the first axis encompassed those of all the individuals that belong to all other Central and Southern Italian breeds, with the exception of the two Maltese populations. These two Maltese populations overlapped each other almost completely but also formed a separate cluster on the left corner of the plot. Only a few MAL and SAM individuals were positioned among the scatter of points for the SAR breed.

**Fig. 4:**
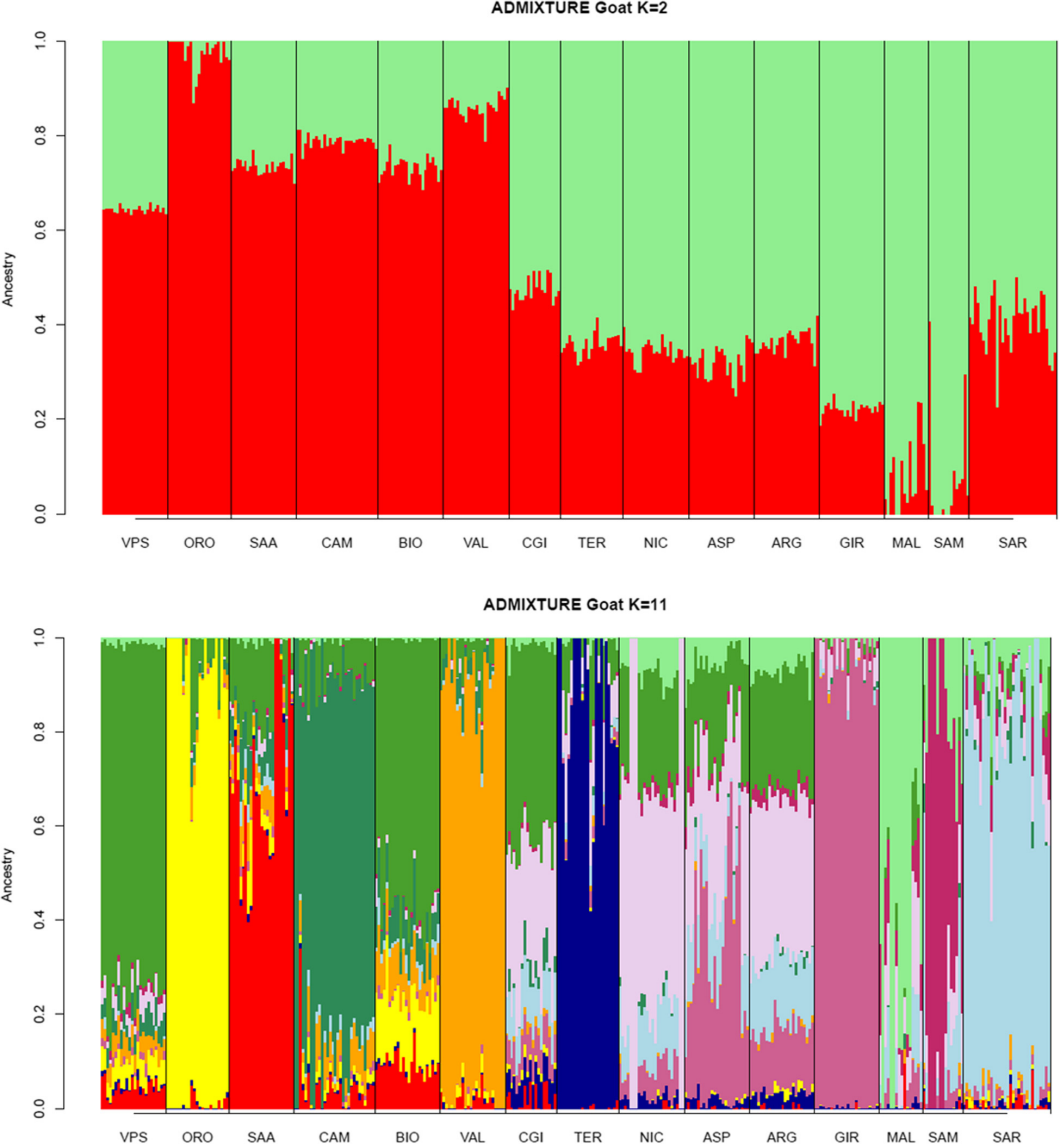
Bayesian clustering performed with ADMIXTURE software on goat genotyping data. Assignment of single individuals (thin vertical bars) to the different clusters when *K* = 2 and *K* = 11 hypothetical populations are assumed. Different colours identify different clusters. The reconstruction at *K* = 11 had the smallest cross-validation error [See Additional file 4 Figure S2]

The Bayesian clustering procedure implemented in ADMIXTURE software at *K* = 2 (Fig. 4) highlighted a differential distribution between breeds from Northern versus Southern Italy. On the one hand, the first component separated the ORO breed with very high Q score values (0.9753 on average) as well as other Northern breeds with average Q scores of 0.7882. On the other hand, the second component showed an opposite trend since it discriminated the two insular Maltese populations (average Q score of 0.9224) and included Central and Southern Italian breeds with an average Q score of 0.6881. When K was increased from 3 to 10 [See Additional file 3: Figure S1 to S8], specific breeds were progressively assigned to distinct clusters: TER at *K* = 3, VAL at *K* = 4, GIR at *K* = 5, the two Maltese populations MAL + SAM at *K* = 6, SAR at *K* = 7, and CAM and SAA at *K* = 8. At *K* = 10, the two Maltese populations were further split into separate clusters. *K* = 11 (Fig. 4) was identified as the best fitting resolution according to the calculation of CV errors [See Additional file 4: Figure S9]. This resolution confirmed the clusters that were progressively revealed at lower K values and also highlighted varying levels of genomic admixture between breeds. The breeds VPS, ORO, SAA, CAM, VAL, TER, GIR, SAR, MAL and SAM were all assigned to different and clearly recognizable clusters. Among the remaining Northern Italian breeds, BIO showed a remarkable level of admixture, with minor components in common with VPS, ORO, VAL and SAA breeds. Except for TER, breeds from Central and Southern Italy (CGI, NIC and ASP) and the Sicilian ARG breed shared a common genomic background of admixed origin. In addition, a genomic component was observed with partial contributions from the gene pools of VPS, GIR and SAR. This confirmed the large overlap in the scatter of points already highlighted for NIC, ARG and ASP breeds in the MDS plot.

## Discussion

Although the 50 K SNP panel was developed from sequence data for goat breeds such as Saanen, Alpine, Creole, Boer, Kacang, and Savanna (http://www.goatgenome.org/), a large number of polymorphic SNPs were detected for the Italian breeds (although not all included in the discovery panel), which suggests that the impact of ascertainment bias is small in our dataset.

The analysis of genotyping data for Italian goats revealed: (i) high levels of genetic polymorphism, (ii) a limited amount of inbreeding, (iii) a geographical pattern that underlies the distribution of genomic diversity, and (iv) a moderate and weak population structure in the Northern and Central-Southern breeds, respectively. The latter two results can also be influenced by traditional management practices, recent demographical events and adaptation to different climatic conditions. However, a more extensive sampling of Italian goat breeds that would cover more evenly the geographical range is necessary to assess the impact of these factors.

### Geographical distribution of Italian goat molecular diversity

The presence of a clear North–south geographical distribution of genetic diversity (Fig. 3) along the longitudinal axis of the Italian Peninsula was highlighted by both the first dimension of the MDS plot and the Neighbour-network reconstruction. A similar geographical pattern has been described in previous studies based on dominant or multiallelic markers in European goats and cattle [11, 28, 29] and on the IlluminaOvineChip50 in Italian sheep breeds [30].

Probably, isolation by geographical distance played a major role in shaping the differentiation of Italian goats, although it may have acted in synergy with other factors. Introgression from gene pools of animals that are native to foreign countries (e.g., Southern Italian breeds may have been crossed with other Southern European or Northern African breeds at the time of the domination of the Arabs) may have also played a role, together with adaptation to local environmental conditions, as already suggested by a genome-wide analysis based on AFLP markers [31]. The effect of geographical isolation at the local scale can be seen in Northern Italian goat breeds that are traditionally reared in the Alps, where geographical barriers such as peaks and steep valleys can strongly limit or prevent gene flow. This is suggested by the barely visible or absent overlap between these breeds on the MDS plot. However, to better clarify the relative roles of all these factors, a comparison with a larger set of European and Mediterranean breeds is necessary.

Although the sizes of the populations analyzed were small, H_E_ values were in line with those calculated for other breeds that are included in the SNP discovery panel [18, 32]. In addition, the difference between H_O_ and H_E_ was not significant for any of the investigated breeds, with the sole exception of NIC for which a slightly positive F_IS_ was found. It is likely that pedigree records and occasional DNA-based controls helped to design mating plans that enabled farmers to control inbreeding. The institution of National Registers and Herd Books (e.g., for CAM in 1973, ORO in 1993, BIO in 1997) further contributed to preserve goat breeds from indiscriminate crossbreeding. This is particularly the case for breeds with distinctive phenotypic traits, such as horn shape in VAL and GIR, and horns and coat colours in the ORO breed.

### Population structure of Italian goats

The analysis of population structure highlighted a moderate tendency for clustering for most of the Northern Italian breeds. This was also confirmed by the MDS plot and can be explained by the demographic history of these breeds that have been reared for a long time in geographically and culturally separate valleys. As a consequence, it is likely that these breeds experienced reproductive isolation and reduced gene flow and thus acquired a strong genetic identity [33]. The effect of cultural and geographical separation due to alpine barriers has already been observed in the genetic makeup of some human populations in the Alps, which still today reflect the topographic features of these mountains [34]. The VPS and BIO breeds are the main exception to this trend with a shared strong genetic component. This is probably due to the geographical proximity of the breeding areas of these two breeds, which facilitates the exchange of bucks and does. As already reported in the literature [10, 28, 35], our results from admixture analysis confirmed the strong genetic identity of the ORO breed: indeed at *K* = 2, this breed is clearly assigned to one of the two gene pools (average Q scores > 0.97; Fig. 4a), and at *K* = 4 [See Additional file 3: Figure S2], all ORO individuals are assigned to a separate gene pool. There are several possible explanations for this situation. The large number of monomorphic SNPs and rare alleles detected for this breed suggests that a strong drift effect has taken place. In fact, the ORO breed, is among the first populations of goats in Northern Italy to have experienced a strong reproductive isolation because of several specific phenotypic traits i.e., four well characterized coat colour patterns that differ from those of breeds in the nearby regions, long hair and a particular shape of horn) and also because of a dramatic demographic decrease of about 90 % in less than 15 years, in spite of the agricultural policy of the European Union (EU) to support native breeds at risk of extinction. Hopefully, new insights on the ORO origin as well as a renewed concern towards its conservation will come from the results of the complete genome sequencing of several unrelated individuals that is currently in progress (unpublished data).

Among the central Italian breeds investigated, TER had the smallest population size, with only 58 animals recorded by the National Breeders Association (www.assonapa.it). Nevertheless, an excess of TER heterozygotes was observed, which, together with the large number of rare SNPs identified for this breed and the wide area covered by TER individuals on the MDS plot, suggest that crossbreeding with bucks from other breeds occurred. However, since admixture analysis assigned TER individuals to a well-defined cluster at a K value as low as 3, which suggests a low level of admixture, if a recent episode of introgression occurred, it probably involved a donor gene pool from a breed that is not included in our dataset.

CGI from Central Italy is also at risk of extinction; in 2008, when the national Register was created, this breed counted only 181 animals that were distributed across a few farms in the Lazio region. Thanks to local (Regolamento (European Council EC) 1698/2005 - Programma di Sviluppo Rurale 2007–2013) and EU economic support, after five years, the population size had increased by 3.7 folds and reached 674 animals in 2013 (www.assonapa.it). The distribution of individual points on the MDS plot and an H_E_ of 0.402 indicate that there is a certain level of genetic variability within this breed. According to admixture analysis, this breed shares a common genomic background with three Southern Italian populations (NIC, ASP and ARG). Although these three breeds are bred in non-contiguous geographical areas - i.e., Southern Lazio for CGI, Calabria for NIC and ASP and Sicily for ARG –, their pairwise F_ST_ distances were the lowest, which can be partly explained by the impact of transhumance in Central and Southern Italy [30] and the trade or occasional exchange of bucks and does between these breeds, which share a similar grey coat colour.

NIC is the only breed that showed a significant F_IS_ value. In 2013, it counted 4975 heads as a result of the adoption in Southern Italy of the Council Regulation (EC) No 1698/2005 to support rural development that includes the presence of autochthonous breeds in danger of extinction; this is funded by the European Agricultural Fund for Rural Development (EAFRD). The absence of male rotational schemes between farms has probably caused inbreeding at the farm level (www.goatit.eu), which shows that conservation strategies that are based exclusively on remuneration of farmers are not efficient when proper breeding schemes and extension services are lacking [36].

Besides demographic factors, historical factors are likely to have an impact on the genetic makeup of Southern Italian and insular breeds. In fact, since the Neolithic age, the area that covers North Africa, Malta, Sicily, Sardinia, and Southern Italy has been deeply interconnected by routes of migration, trade and conquest. The tightly interconnected history of these areas was favoured by their role of crossroads of the trading and warring routes through the land-locked Mediterranean Sea [37].

The differentiation between Maltese goats that were sampled in Sardinia and Sicily as revealed by admixture and Neighbour-network analyses, is probably the consequence of the combined effects of genetic drift, small population size, founder effects and reproductive isolation (since the Southern Tyrrhenian Sea acted as a strong genetic barrier). However, the two populations were not separated on the MDS plot based on a genomic kinship distance matrix. In fact, this measure may be showing that these two populations share a common origin since it can account for older evolutionary relationships and is less affected by recent population dynamics [38]. Anyway, the ancestral origin of the Maltese breed as a whole remains uncertain and previous studies suggested that the distant roots of this breed may be in the Middle-Eastern side of the Mediterranean basin and that it probably derives from crosses between North African and Italian goats [39, 40]. To test this hypothesis, it is necessary to compare the MAL breed with a larger set of goat breeds from the Mediterranean area, Northern Africa and Spain.

## Conclusions

According to our results, the axis of the main source of genetic variation for Italian goat populations stretches along the longitudinal axis of the Italian peninsula. Among the major factors which could have acted on the goat genome in Italy, reproductive isolation due to geographical distance, adaptation to local conditions and breeders’ management may all have a role, although the identification of their relative impacts and contribution is not straightforward. To assess the importance of any of these factors, a larger and more comprehensive set of breeds is necessary, in particular to have a more uniform distribution of sampling locations. Nevertheless, some breeds displayed a clear genetic identity that confirms previous findings [9–11, 28, 35], although its source is not always completely understood e.g., the ORO breed. The adoption of European or National conservation policies has boosted the population size of breeds at risk of extinction and probably affected the recent evolutionary history of goat populations in Italy. However, the lack of extensive records on demographic trajectories makes it difficult to either confirm or challenge this hypothesis. Based on these observations, it is clear that understanding the extent, distribution and origin of present-day genetic diversity is a complex task that requires other sources of information than molecular data only.

A thorough genomic characterization of breeds represents a key point to develop efficient conservation strategies, which, to become effective, should also take into account population viability. This variable, in turns, depends on budgetary limitations, management practices and on the existence of services to support shepherds.

Based on the use of a standardized genotyping array, such as the GoatSNP50 BeadChip, it will be possible to combine various datasets and to provide a global picture of goat genetic diversity both at a local and global scale. This will help to understand the origins of genetic diversity and to manage biodiversity of these animal genetic resources that are particularly relevant for poor and marginal rural areas of the world.

## Additional files

Additional file 1: Table S1. Upper triangular matrix of pairwise F_ST_ index values (all statistically significant at *P* < 0.001). Description: F_ST_ distance matrix between breeds calculated by Arlequin software ver. 3.5.1.3. Additional file 2: Table S2. AMOVA results: between breeds (upper part) and between groups of breeds from different geographical areas (i.e., Northern Italy, Central Italy, Southern Italy and islands) (lower part). Description: Analysis of MOlecular VAriance (AMOVA) at different hierarchical levels to test the differentiation between breeds and between groups of breeds from distinct geographical areas (i.e., Northern Italy, Central Italy, Southern Italy and islands). Additional file 3: Figures S1 to S8. Title: Bayesian clustering performed with ADMIXTURE software on goat SNP data. Figure S1, *K* = 3; Figure S2, *K* = 4; Figure S3, *K* = 5; Figure S4, *K* = 6; Figure S5, *K* = 7; Figure S6, *K* = 8; Figure S7, *K* = 9; Figure S8, *K* = 10. Description: Results of population structure analysis with a number of hypothetical pseudo-populations, K, varying from 3 to 10 obtained by ADMIXTURE ver. 1.22 software. Additional file 4: Figure S9. Cross-validation errors calculated for ADMIXTURE software analysis at K values ranging from 2 to 25. Description: Cross-validation (CV) error values were computed for each K using a 5-fold cross validation procedure.
