# Supplementary material for: Genetic diversity of Italian goat breeds assessed with a medium-density SNP chip
Source: Genet Sel Evol. 2015 Aug 4;47(1):62. doi: 10.1186/s12711-015-0140-6 (PMC4523021; doi:10.1186/s12711-015-0140-6)
Supplement: Additional file 1: Table S1. — Upper triangular matrix of pairwise FST index values (all statistically significant at P < 0.001). Description: FST distance matrix between breeds calculated by Arlequin software ver. 3.5.1.3. [file 12711_2015_140_MOESM1_ESM.docx]

Table S1

|  | **VAL** | **CAM** | **SAA** | **ORO** | **BIO** | **VPS** | **CGI** | **TER** | **ASP** | **NIC** | **ARG** | **GIR** | **MAL** | **SAR** | **SAM** |
| --- | --- | --- | --- | --- | --- | --- | --- | --- | --- | --- | --- | --- | --- | --- | --- |
| **VAL** | — | 0.064 | 0.067 | 0.113 | 0.058 | 0.064 | 0.072 | 0.140 | 0.078 | 0.081 | 0.071 | 0.125 | 0.133 | 0.076 | 0.140 |
| **CAM** |  | — | 0.047 | 0.096 | 0.044 | 0.048 | 0.054 | 0.120 | 0.061 | 0.063 | 0.054 | 0.107 | 0.112 | 0.059 | 0.117 |
| **SAA** |  |  | — | 0.092 | 0.038 | 0.042 | 0.048 | 0.114 | 0.056 | 0.058 | 0.049 | 0.102 | 0.106 | 0.057 | 0.113 |
| **ORO** |  |  |  | — | 0.071 | 0.084 | 0.094 | 0.162 | 0.101 | 0.103 | 0.093 | 0.147 | 0.156 | 0.103 | 0.164 |
| **BIO** |  |  |  |  | — | 0.028 | 0.036 | 0.104 | 0.044 | 0.046 | 0.036 | 0.091 | 0.098 | 0.049 | 0.105 |
| **VPS** |  |  |  |  |  | — | 0.032 | 0.101 | 0.040 | 0.042 | 0.033 | 0.087 | 0.093 | 0.049 | 0.101 |
| **CGI** |  |  |  |  |  |  | — | 0.090 | 0.028 | 0.029 | 0.020 | 0.077 | 0.080 | 0.039 | 0.090 |
| **TER** |  |  |  |  |  |  |  | — | 0.095 | 0.095 | 0.087 | 0.143 | 0.146 | 0.105 | 0.156 |
| **ASP** |  |  |  |  |  |  |  |  | — | 0.025 | 0.013 | 0.045 | 0.069 | 0.037 | 0.078 |
| **NIC** |  |  |  |  |  |  |  |  |  | — | 0.015 | 0.076 | 0.066 | 0.038 | 0.077 |
| **ARG** |  |  |  |  |  |  |  |  |  |  | — | 0.062 | 0.060 | 0.029 | 0.071 |
| **GIR** |  |  |  |  |  |  |  |  |  |  |  | — | 0.126 | 0.086 | 0.133 |
| **MAL** |  |  |  |  |  |  |  |  |  |  |  |  | — | 0.070 | 0.061 |
| **SAR** |  |  |  |  |  |  |  |  |  |  |  |  |  | — | 0.075 |
| **SAM** |  |  |  |  |  |  |  |  |  |  |  |  |  |  | — |
