# Supplementary material for: Genetic diversity of Italian goat breeds assessed with a medium-density SNP chip
Source: Genet Sel Evol. 2015 Aug 4;47(1):62. doi: 10.1186/s12711-015-0140-6 (PMC4523021; doi:10.1186/s12711-015-0140-6)
Supplement: Additional file 2: Table S2. — AMOVA results: between breeds (upper part) and between groups of breeds from different geographical areas (i.e., Northern Italy, Central Italy, Southern Italy and islands) (lower part). Description: Analysis of MOlecular VAriance (AMOVA) at different hierarchical levels to test the differentiation between breeds and between groups of breeds from distinct geographical areas (i.e., Northern Italy, Central Italy, Southern Italy and islands). [file 12711_2015_140_MOESM2_ESM.docx]

Table S2

| Source of variation | Sum of squares | Variance components | Percentage variation |
| --- | --- | --- | --- |
| Among populations | 611816.06 | 737.28 | 7.49 |
| Among individuals within populations | 3140414.41 | 264.19 | 2.68 |
| Within individuals | 3096094.00 | 8845.46 | 89.83 |
| Total | 6848324.47 | 9847.46 |  |
| Among groups | 180618.59 | 233.53 | 2.11 |
| Among populations within groups | 502222.46 | 681.06 | 6.16 |
| Within populations | 6935333.79 | 10139.56 | 91.73 |
| Total | 7618174.84 | 11054.15 |  |
