# Supplementary figures and images for: Genetic diversity of Italian goat breeds assessed with a medium-density SNP chip
Source: Genet Sel Evol. 2015 Aug 4;47(1):62. doi: 10.1186/s12711-015-0140-6 (PMC4523021; doi:10.1186/s12711-015-0140-6)

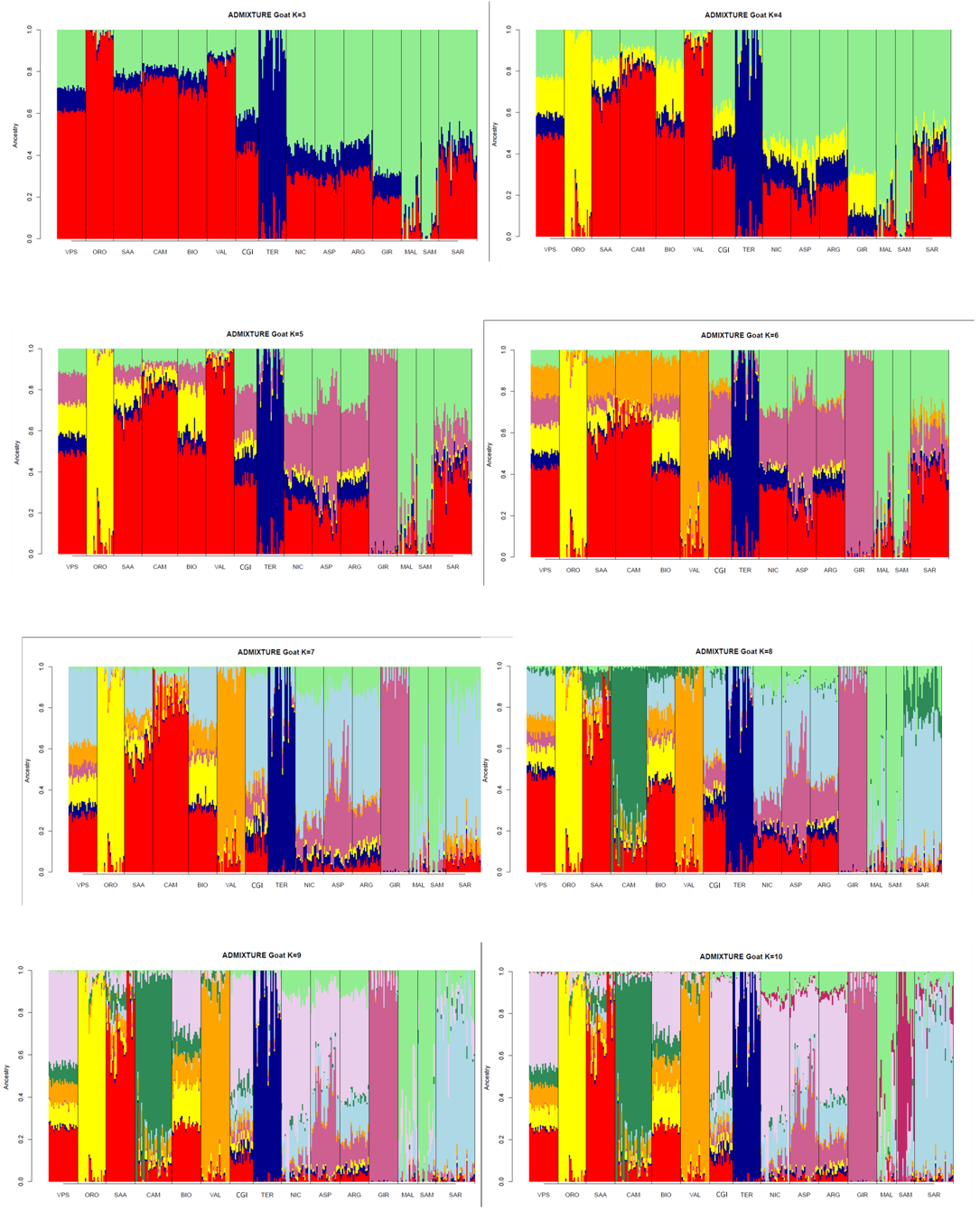

Supplement: Additional file 3: Figures S1 to S8. — Title: Bayesian clustering performed with ADMIXTURE software on goat SNP data. Figure S1, K = 3; Figure S2, K = 4; Figure S3, K = 5; Figure S4, K = 6; Figure S5, K = 7; Figure S6, K = 8; Figure S7, K = 9; Figure S8, K = 10. Description: Results of population structure analysis with a number of hypothetical pseudo-populations, K, varying from 3 to 10 obtained by ADMIXTURE ver. 1.22 software. [file 12711_2015_140_MOESM3_ESM.png]

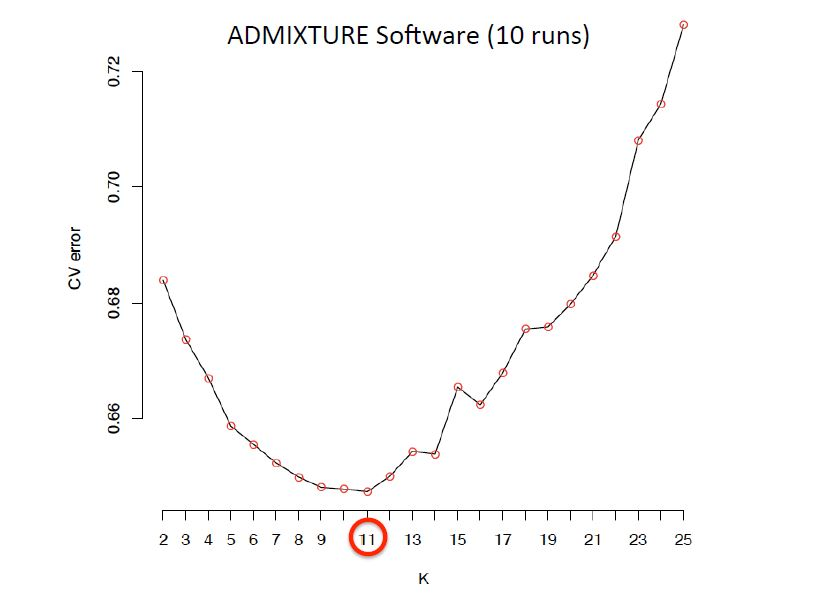

Supplement: Additional file 4: Figure S9. — Cross-validation errors calculated for ADMIXTURE software analysis at K values ranging from 2 to 25. Description: Cross-validation (CV) error values were computed for each K using a 5-fold cross validation procedure. [file 12711_2015_140_MOESM4_ESM.png]
